# Whole-exome sequencing reveals genetic etiology and pregnancy outcomes in fetuses with ultrasound-detected abnormalities at a tertiary center

**DOI:** 10.3389/fgene.2026.1858421

**Published:** 2026-10-13

**Authors:** Meiying Cai, Na Lin, Hailong Huang, Meimei Fu, Yifang Dai, Liangpu Xu

**Affiliations:** Fujian Key Laboratory for Prenatal Diagnosis and Birth Defect, Medical Genetic Diagnosis and Therapy Center, Fujian Maternity and Child Health Hospital College of Clinical Medicine for Obstetrics & Gynecology and Pediatrics, Fujian Medical University, Fuzhou, China

**Keywords:** etiology, fetus, phenotype, ultrasound, whole-exome sequencing

## Abstract

Despite advances in prenatal diagnostics, the genetic causes underlying fetal ultrasound abnormalities remain poorly understood, with a significant proportion of cases lacking definitive molecular diagnoses. Invasive prenatal testing was performed in 179 cases, followed by whole-exome sequencing (WES) to identify genetic etiologies. Among 179 abnormal fetuses, 132 had single-system and 47 had multisystem abnormalities. WES identified pathogenic (P) or likely pathogenic (LP) variants in 33 cases (19 single-system, 14 multisystem), with skeletal (7), cardiac (5), and craniofacial defects (2) being most frequent. Thirty-four genes were involved, encompassing 41 variants (15 missense, 10 frameshift, 6 splicing, 6 nonsense, and 4 in-frame deletions). WES yielded a P/LP rate of 17.88% (32/179) for variants that explained the fetal phenotype, with an additional pathogenic finding (overall P/LP rate: 18.44%, 33/179). Inheritance patterns were autosomal dominant (19), recessive (12), X-linked (1), and mosaic (1). Family studies confirmed 27 variants (18 inherited, 9 *de novo*). Pregnancy outcomes included 60 terminations, 19 intrauterine deaths, and 8 postnatal abnormalities. Most fetal ultrasound phenotypes matched the disease-causing gene profiles. Combining molecular testing with ultrasound helps clinicians identify genetic causes, assess recurrence risk, and inform parents, enabling personalized management for high-risk families.

## 1 Introduction

An increasing number of fetal ultrasound abnormalities have been diagnosed owing to recent improvements in ultrasound technology. Congenital developmental abnormalities occur in approximately 3% of full-term newborns (Huang W. et al., 2023; He et al., 2021). Fetal abnormalities detected using prenatal ultrasonography are often accompanied by chromosomal abnormalities, gene variants, and partial phenotypes of certain genetic syndromes. Determining the relationship between fetal ultrasound abnormalities and genetics is crucial for improving patient prognosis and clinical decision making (Mone et al., 2021; Diderich et al., 2021). Prenatal diagnosis of fetal ultrasound abnormalities requires invasive procedures, such as chromosome karyotyping and chromosomal microarray analysis (CMA). The former is widely used but can only detect large fragment deletions and duplications of > 10 Mb, among other limitations. In comparison, CMA performs better in detecting fetal ultrasound abnormalities by up to 6%–9% (Chou et al., 2022; Lin et al., 2022; Levy and Wapner, 2018; Hu et al., 2020). Approximately 20% of fetuses with ultrasound abnormalities are diagnosed using a combination of these techniques (Petrovski et al., 2019). Unfortunately, the genetic causes are not confirmed in >80% of cases, promoting the risk of death or disability after birth and limiting pregnancy management.

High-throughput whole-exome sequencing (WES) technology has recently been applied to detect congenital diseases, greatly improving the diagnostic accuracy for genetic diseases and garnering attention in prenatal diagnostics in China and abroad (Diderich et al., 2021; Reilly et al., 2024; Xiang et al., 2024; Jin et al., 2024; Qin et al., 2023; Yates et al., 2017). WES can detect genetic diseases related to gene variation at the single-base level and is a supplementary method for characterizing fetal ultrasound abnormalities without a clear etiology based on the CMA (Petracchi et al., 2019). In our diagnostic pipeline, WES was employed as a complementary, secondary test following without pathogenic (P) or likely pathogenic (LP) variants from karyotyping and/or CMA. It was not used as a first-tier test. This reflexive testing framework is critical for interpreting the diagnostic yield reported in this study.

This WES study assessed the genetic characteristics of 179 fetuses showing ultrasound abnormalities. This study provides a useful framework for high-throughput prenatal genetic diagnosis and efficient consultations.

## 2 Methods

### 2.1 Patient data

From January 2022 to December 2023, 179 pregnant women (aged 19–44 years, gestational week 11–36 ± 6) with abnormal fetal ultrasound findings were selected for prenatal diagnosis at the medical genetic diagnosis center of the fujian maternity and child health care hospital (Fuzhou, China). The gestational ages at the time of testing ranged from 11 to 36 weeks, with cases distributed as follows: 95.53% (171/179) in the second trimester (14–27+6 weeks), 3.35% (6/179) in the third trimester (≥ 28 weeks), and 1.12% (2/179) in the first trimester (<14 weeks).

All participants visited the prenatal diagnostic clinic for consultation and underwent prenatal villi or amniotic fluid extraction for WES testing. The inclusion criteria were as follows: nervous system abnormalities, nuchal translucency thickening, edema, congenital heart defects, congenital malformations of the urinary system, skeletal deformities, multiple abnormalities, digestive system malformations, craniofacial deformities, and other abnormalities. The exclusion criterion was normal intrauterine ultrasound findings. All cases were referred to our tertiary fetal medicine center following the detection of structural anomalies or soft markers on a detailed prenatal ultrasound. None of the patients had clinically significant aneuploidies or P/LP variants detected by standard karyotyping and/or CMA, which preceded WES.

### 2.2 Genetic counseling and informed consent process

All families received formal, standardized pre-test genetic counseling provided by a board-certified genetic counselor and / or clinical geneticist. This session covered the purpose, potential outcomes (including diagnostic findings, variants of uncertain significance, and the possibility of incidental/secondary findings), limitations of the test, potential psychological impacts, and reproductive options based on possible results. Written informed consent was obtained from all participating families following the counseling session. The consent form specifically addressed the scope of analysis, data handling, and the possibility of receiving different types of results. All results, whether diagnostic or non-diagnostic, were disclosed by the clinical team (geneticist and genetic counselor) in a dedicated post-test counseling session. This session focused on interpreting the results in the context of the fetal phenotype, discussing prognosis, reviewing reproductive options and recurrence risks, and providing psychosocial support.

### 2.3 Intrauterine fetal ultrasound

A GE Voluson E8 ultrasound Doppler system and supporting probes were used to examine the fetal brain, spine, face, neck (sagittal, transverse, and coronal sections), heart and great vessel plane, thoracic cavity, abdominal wall and cavity plane, urinary system, skeletal system, and tumor. Three typical images were obtained from each standard section. All data were stored on an ultrasound workstation.

### 2.4 Genomic DNA extraction and WES

Data regarding participant age, ultrasound findings, and full genetic consultation were acquired with their consent, and fetal villi or amniotic fluid samples were collected. The villi samples were washed with sterile normal saline and stored in glass bottles containing normal saline. Fetal DNA was extracted from 10 mL of fully cultured amniotic fluid or cells obtained from tissues treated with pancreatic enzymes under sterile conditions using a DNA extraction kit (QIAGEN, Hilden, Germany). Fetal DNA was first fragmented, and a library was then prepared. The DNA of the target gene exon and adjacent cut region was captured and enriched using a Roche KAPA HyperExome chip (Roche, Nutley, NJ, United States), and mutations were detected using an Illumina second-generation sequencing platform. The data quality control indicators were as follows: average sequencing depth of the target region ≥ 100 × , and > 95% of sites had an average depth of > 20×. GRCh37 (hg19) was selected as the human reference genome to maintain consistency with our laboratory’s historical datasets and standard variant annotation pipelines. Short-read sequences were aligned to the GRCh37 (hg19) human reference genome using SOAPaligner and BWA (v0.7.17; Li Heng, Wellcome Trust Sanger Institute, Cambridge, United Kingdom), followed by duplicate read removal. SOAPsnp was developed by BGI, Shenzhen, Guangdong, China; GATK (v4.2.0.0) was obtained from the Broad Institute, Cambridge, MA, United States. Exon-level copy number variants were identified with ExomeDepth (v1.1.16). Variant annotation was performed via VEP (Variant Effect Predictor, v110). The Ensembl Variant Effect Predictor was used to annotate SNPs/InDels, including all transcripts in the Ensembl, RefSeq, and CCDS databases. Public allele frequency annotations, including 1,000 Genomes (1 KG), ESP, HapMap, and Exome Aggregation Consortium, were added to a hazard or conservative prediction tool score, including SIFT, Mutation Taster, LRT, PhastCons, PolyPhen2-HDIV, PolyPhen2-HVAR, Mutation Assessor, FATHMM, GERP++, PhyloP, and GERP. Mutations in healthy individuals were filtered using the 1,000 Genomes Project, Genome Aggregation Database, Exome Aggregation Consortium, ClinVar, dbSNP, Human Phenotype Ontology, OMIM, MalaCards, and Human Gene Mutation and Human Genome databases. The allele frequencies of healthy populations in the Variation Society, UCSC Genome Browser, DECIPHER, and NCBI Genome were screened to retain low-frequency rare variations. Non-pathogenic variants were filtered. Variants were classified according to the American College of Medical Genetics and Genomics Standards and Guidelines (Richards et al., 2015). Pathogenic or suspected pathogenic variations were verified using Sanger sequencing. Variants of uncertain significance (VUS) were not reported as diagnostic but were documented in the laboratory report. Only P/LP variants were considered positive findings for diagnostic yield calculation and clinical reporting. The study employed a singleton (proband-only) WES design. WES was performed on the fetal DNA sample. Identified candidate variants were subsequently verified and assessed for inheritance through targeted Sanger sequencing in the parental samples. This targeted familial segregation analysis, rather than trio-based WES, was the basis for assigning variants as *de novo* or inherited. The degree of maternal contamination was routinely quantified using targeted PCR analysis of highly polymorphic short tandem repeat (STR) markers, comparing fetal and maternal DNA profiles. This quantitative assessment was a standard part of our quality control pipeline. Variants were classified according to the ACMG/AMP guidelines by two independent clinical laboratory geneticists. Any discordant classifications were discussed in a multidisciplinary team meeting involving clinical geneticists, molecular pathologists, and bioinformaticians to reach a final consensus classification.

### 2.5 Follow-up

All cases were followed up for > 6 months, and pregnancy outcomes and fetal development were recorded, among other clinical information.

### 2.6 Statistical analysis

SPSS 25.0 software (IBM SPSS, Armonk, NY, United States) was used to analyze the count (percentage) [n (%)] data. The chi-square test was used for comparisons between groups, with a statistical significance set at *P* < 0.05. All 95% confidence intervals for binomial proportions of P/LP variant detection rates were calculated using the exact Clopper-Pearson method, which provides appropriate statistical uncertainty estimates for small sample sizes, sparse counts, and zero-event subgroups.

## 3 Results

### 3.1 Classification of phenotypic abnormalities in intrauterine fetal ultrasound

Among the 179 fetuses with ultrasound abnormalities, 132 (73.74%, 132/179) had single-system abnormalities, 37 of which were skeletal deformities that primarily manifested as short limbs, abnormal spine development, and fetal growth restriction (Table 1). Twenty-nine patients presented with congenital heart defects, including ventricular septal defects, aortic arch, and pulmonary artery stenosis. Twenty-six patients presented with an abnormal urinary system, with the main manifestations being dysplastic kidney, hyperechogenic kidney, and pyelectasis. An abnormal nervous system was observed in 13 patients, mainly characterized by hydrocephalus and corpus callosum dysplasia, and three patients showed craniofacial deformity, mainly cleft lip and palate. Digestive system malformations were found in four patients, mainly presenting as intestinal obstruction. Nuchal translucency thickening, edema, and polyhydramnios was observed in 11, 5, and 4 patients, respectively. Multiple systemic abnormalities were observed in 47 patients (26.26%, 47/179) (Figure 1).

**FIGURE 1:**
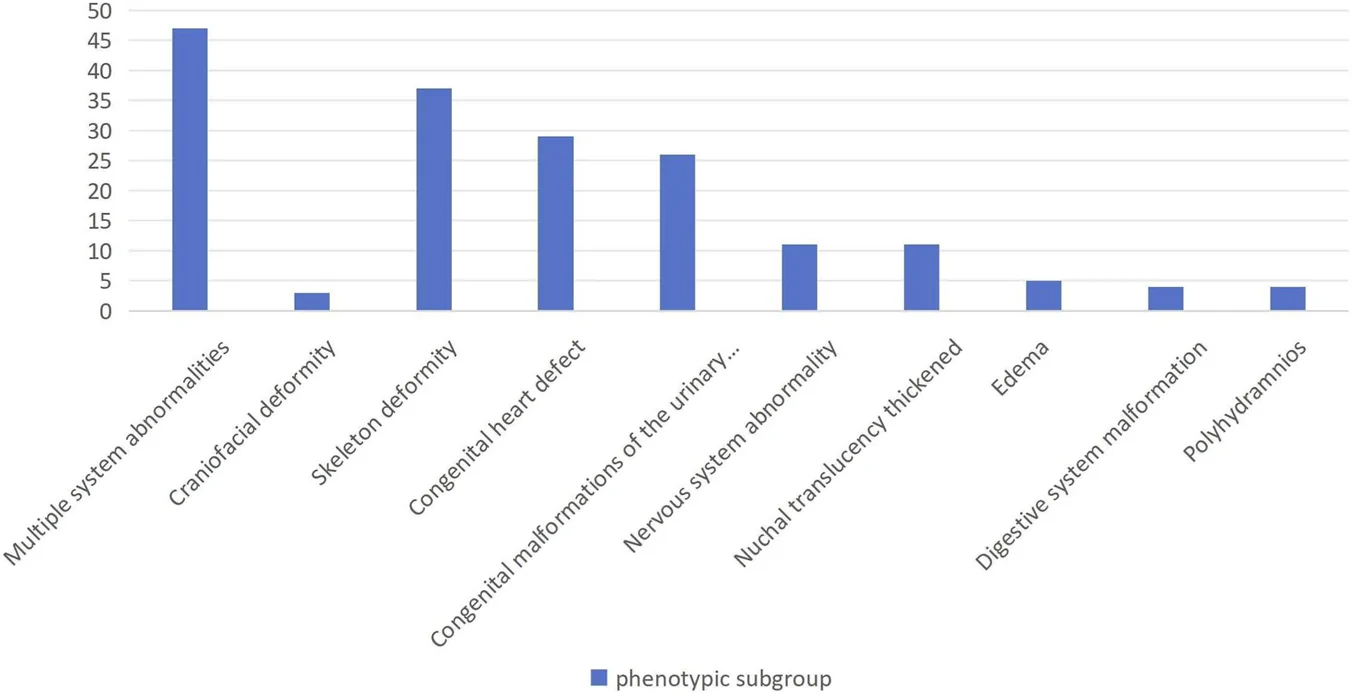
Phenotype distribution.

**TABLE 1:** Classification of fetuses with abnormal intrauterine ultrasound.

Abnormal intrauterine ultrasound	Number of cases
Multiple system abnormalities	47
Skeleton deformity	37
Congenital heart defect	29
Congenital malformations of the urinary system	26
Nervous system abnormality	13
Craniofacial deformity	3
Digestive system malformation	4
Nuchal translucency thickened	11
Edema	5
Polyhydramnios	4

### 3.2 WES results for phenotypic abnormalities in intrauterine fetal ultrasound

WES was performed on 179 fetal samples, resulting in a primary phenotypic diagnosis in 32 cases (17.88%). In addition, one incidental finding unrelated to the fetal phenotype was detected. Therefore, clinically significant P/LP variants were identified in a total of 33 cases (18.44%). Among the 33 fetuses with P/LP variants, 19 exhibited single-system abnormalities (19/132, 14.39%), and 14 had multisystem abnormalities (14/47, 29.79%). The WES detection rates varied significantly between the groups (*P* = 0.02). Among the fetuses with P/LP variants, the most common ultrasound abnormalities were craniofacial deformities (2/3, 66.67%), skeletal deformity (7/37, 18.92%), congenital heart defects (5/29, 17.24%), nervous system (15.38%; 2/13), and congenital malformations of the urinary system (11.54%; 3/26). No P/LP variants were detected in fetuses with digestive system malformations, thickened nuchal translucency, edema, or polyhydramnios (Figure 2).

**FIGURE 2:**
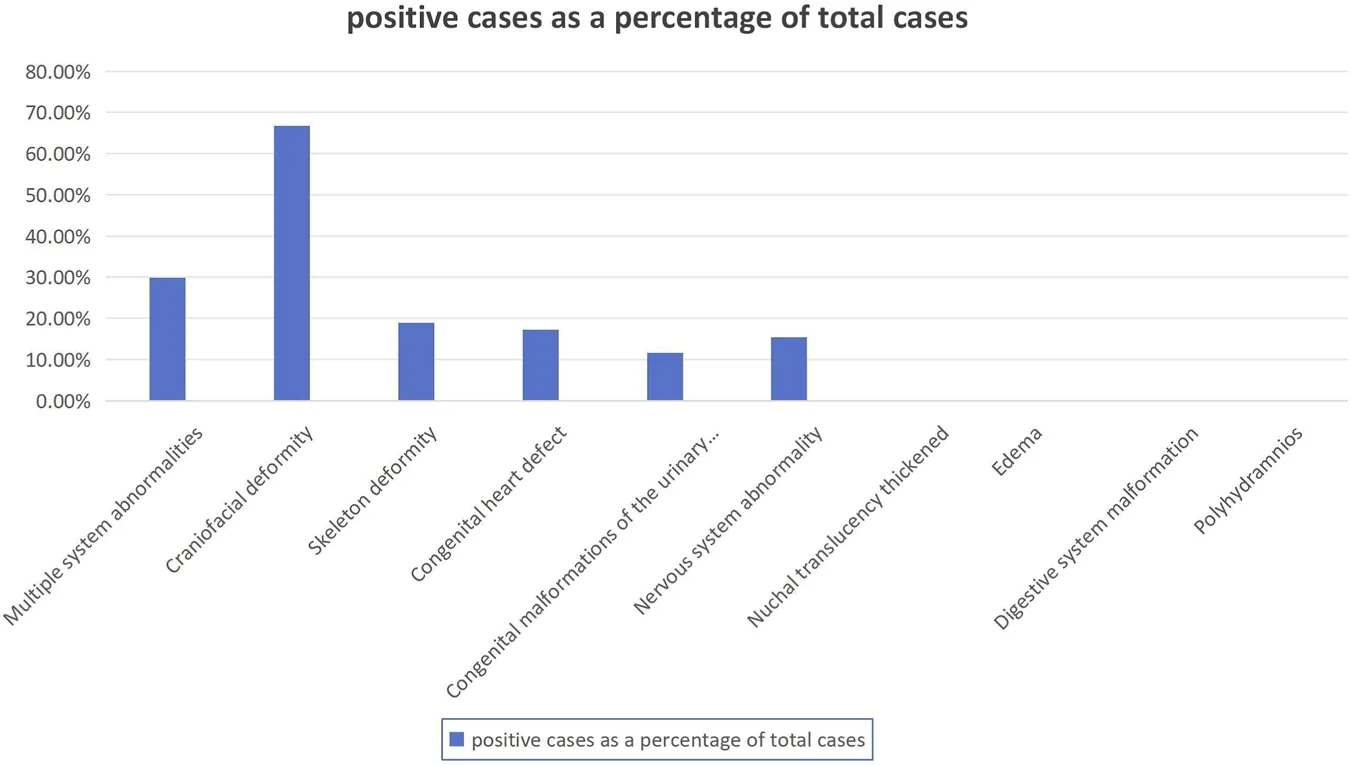
Diagnostic efficiency.

### 3.3 Mutation types and inheritance patterns of genetic variation

Among the cases with P/LP variants (33/179), 34 genes were detected, along with 41 pathogenic variants, including 15 missense, 10 frameshift, 6 splicing, 6 nonsense, and 4 in-frame deletions (Table 2). Compound heterozygous variants were detected in eight patients. Among the 33 patients, 19 (57.58%, 19/33) showed autosomal dominant (AD) inheritance, 12 (36.36%, 12/33) autosomal recessive (AR) inheritance, 1 (3.03%, 1/33) X-linked recessive inheritance, and 1 (3.03%, 1/33) had mosaic abnormalities. Of the 19 patients with AD inheritance, 8 were associated with *de novo* variations, 6 with heredity, and 5 were unknown. After adequate genetic counseling, the parents of six fetuses refused to undergo family verification, resulting in an unknown genetic mode of inheritance. Among the 27 verified cases, mutations in 18 (66.67%) were inherited from the parents, and 9 (33.33%, 9/27) were *de novo*. In total, nine *de novo* variants were identified across our cohort, comprising eight *de novo* variants linked to autosomal dominant conditions and one separate *de novo* mosaic AKT1 variant. Variant classification was performed according to ACMG/AMP guidelines. Pathogenicity was assessed using population databases (e.g., gnomAD), *in silico* prediction tools, and available literature. Inheritance patterns (e.g., *de novo*, parental origin) were determined using familial segregation data when available, and considered as supporting evidence (e.g., PS2, PP1) under ACMG criteria. For cases involving reduced penetrance or unaffected carrier parents, this information was explicitly noted in the classification. The presence of an unaffected carrier does not automatically rule out pathogenicity for known incompletely penetrant disorders, and such variants were classified accordingly with a comment on penetrance.

**TABLE 2:** Ultrasound phenotype, gene mutation, and pregnancy outcomes of 33 fetuses with P/LP variants.

Case	Ultrasound phenotype	Gene	Variation	Heterozygosity	Mode of inheritance	ACMG variation classification	Source of variation	Related diseases	Pregnancy outcome
1	Cleft lip, cleft palate, oral cleft	FGF8	c.559_573del p.R187_G191del	Het	AD	P (PS3,PM4 PM2_Supporting)	Paternal (het)	Hypogonadotropic hypogonadism 6 with or without anosmia	TP
2	Intrauterine growth retardation	OBSL1	c.458dupGp.L154Pfs*100	Hom	AR	P (PVS1, PM3_Strong,PM2_Supporting)	Paternal/Maternal (het)	3-M syndrome 2	TP
3	Intracranial multiple hyperechoic nodules, cardiac rhabdomyoma, cortical tubercle	TSC2	c.1750_1754dup p.V586Rfs*114	Het	AD	P (PVS1, PS2, PM2_Supporting)	Denovo	Tuberous sclerosis-2, focal cortical dysplasia, type II, somatic, lymphangioleiomyomatosis, somatic	Intrauterine fetal death
4	Cataract, subependymal cysts	AGK	c.424–2A > C c.1047-1G > T	Het Het	AR	P (PVS1 PM2_Supporting) P (PVS1,PM2_Supporting)	Paternal (het) Maternal (het)	Sengers syndrome, cataract 38, autosomal recessive	Intrauterine fetal death
5	Tetralogy of fallot, tricuspid regurgitation, ventricular hypertrophy, pericardial effusion	PLD1	c.2197C > T p.Q733* c.1171C > T p.R391C	Het Het	AR	P (PVS1, PP4 PM2_Supporting) P (PM3_Strong, PP4,PM2_Supporting)	Maternal (het) Paternal (het)	Cardiac valvular dysplasia 1	Intrauterine fetal death
6	Renal cyst	PKD1	c.12391_12393del p.E4131del	Het	AD	P (PM1, PM4, PP4,PP1_Moderate,PM2_Supporting)	Unknown	Polycystic kidney disease 1	Normal phenotype
7	Hydrocephalus, inferior vermis hypoplasia	COL4A1	c.1181G > A p.G394D	Het	AD	P (PM2_Supporting, PP3)	Paternal (het)	Brain small vessel disease with or without ocular anomalies	Intellectual disability
8	Thickened nuchal skin Fold, talipes equinovarus, flexion contracture, pleural effusion	SNAP25	c.593G > C p.R198P	Het	AD	P (PS2_VeryStrong, PM1, PM2_Supporting)	Denovo	Myasthenic syndrome, congenital	TP
9	Intrauterine growth retardation	DNMT3A	c.541C > T p.R181C	Het	AD	P (PS2, PP3 PM2_Supporting)	Denovo	Heyn-sproul-jackson syndrome, atton-Brown-rahman syndrome	TP
10	Ventriculomegaly, renal cortical cysts, hyperechogenic kidneys, Dilatation of the renal pelvis, oligohydramnios	KMT2D	c.6547_6560del p.Y2183Pfs*14	Het	AD	P (PVS1, PS2,PM2_Supporting	Denovo	Kabuki syndrome 1	TP
11	Atrial septal defect, abnormal heart morphology	DUOX2	c.1588A > T p.K530*	Hom	AR	P (PVS1, PM3_VeryStrong)	Paternal/Maternal (het)	Thyroid dyshormonogenesis 6	TP
12	Intrauterine growth retardation, aplasia of the nasal bone	SOX5	c.501delA p.K167Nfs*22	Het	AD	P (PVS1,PM2_Supporting)	Maternal (het)	Lamb-shaffer syndrome	TP
13	Overgrowth, increased head circumference, abnormality of neuronal migration, abnormal morphology of the great vessels, dilation oflateralventricles, thin corpus callosum	AKT1	c.49G > A p.E17K	Mosaic (51/147)	-	P (PS2, PS3, PS4,PM2_Supporting)	Denovo	*Proteus* syndrome, somatic, cowden syndrome 6	TP
14	Cleft palate, thickened nuchal skin fold, decreased head circumference, talipesequinovarus	EFTUD2	c.1304_1305 delTG p.V435Afs*15	Het	AD	P (PVS1, PS2,PM2_Supporting)	Denovo	Mandibulofacial dysostosis Guion-almeida type	TP
15	Hydrops fetalis, pericardial effusion, right atrial enlargement, oligohydramnios, talipes, abnormal heart morphology	HBA1 HBA2	--SEA/--SEA	Hom	AR	P (PVS1, PS4, PM3_Supporting)	Unknown	Thalassemias, alpha-	Intrauterine fetal death
16	Cleft lip, cleft palate, orofacialcleft	CREBBP	c.4614delC p.Y1539Ifs*11	Het	AD	P (PVS1 PS2_Moderate PM2_Supporting)	Denovo	Rubinstein-taybi syndrome 1	Intrauterine fetal death
17	Short long bone, limb undergrowth, intrauterine growth retardation	FGFR3	c.1118A > G p.Y373C	Het	AD	P (PS2_VeryStrong, PS3, PS4, PM2_Supporting, PP3)	Unknown	Achondroplasia Crouzon syndrome with acanthosis nigricans, hypochondroplasia	Intrauterine fetal death
18	Flexioncontracture, fetal akinesia sequence)	ALPL	c.532T > C p.Y178H c.655A > G p.M219V	Het Het	AR	P (PM3_Strong, PS3_Supporting,PM2_Supporting, PP1) P (PM3_Strong PS3_Supporting, PM2_Supporting,PP3)	Paternal (het) Maternal (het)	Hypophosphatasia, childhood, hypophosphatasia, infantile, hypophosphatasia, adult	Intrauterine fetal death
19	Microcephaly, cortical dysplasia, abnormal cortical gyration, abnormality of neuronal migration, aplasia/Hypoplasia of the cerebellar vermis)	MCPH1	c.233 + 2T > G c.229G > T p.E77*	Het Het	AR	P (PVS1,PM2_Supporting) P (PVS1,PM2_Supporting)	Paternal (het) Maternal (het)	Microcephaly 1, primary, autosomal recessive	TP
20	Polyhydramnios, hydrops	SLC12A1	c.1411C > T p.R471*	Het	AR	P (PVS1, PM3,PM2_Supporting)	Maternal (het)	Bartter syndrome, type 1	TP
21	Aplasia/Hypoplasia of the cerebellum, talipes	PTPN11	c.182A > G p.D61G	Het	AD	P (PS2_VeryStrong,PS4_Moderate,PS3_Supporting,PM2_Supporting, PM5, PP3)	Denovo	Noonan syndrome 1	TP
22	Dilatation of the renal pelvis, echogenic intracardiac focus, aplasia of the gallbladder	EPHB4	c.2590C > T p.R864W	Het	AD	P (PS2,PS3_Supporting,PM2_Supporting, PP3)	Denovo	Lymphatic malformation 7, capillary malformation-arteriovenous malformation 2	TP
23	Truncus arteriosus	ZIC3	c.102_124del p.M35Lfs*87	Het	XLR	P (PVS1, PM2)	Maternal	Congenital heart defects, nonsyndromic, 1, X-linked, heterotaxy, visceral, 1, X-linked	Normal phenotype
24	Fetal bowel dilatation, echogenic fetal bowel, abnormal bladder morphology, anal atresia, cloacal abnormality	GREB1L	c.2913delC p.K972Rfs*38	Het	AD	P (PVS1,PM2_Supporting)	Paternal (het)	Renal hypodysplasia/aplasia	Intrauterine fetal death
25	Polycystic kidney, oligohydramnios	BBS1	c.1177C > T (p.A rg393*)	Hom	AR	PVS1+PM2+PM3_Supporting	Paternal/Maternal (het)	Bardet-biedl syndrome 1	TP
26	Hyperechogenic Kidneys, enlarged kidneys, oligohydramnios	BBS2	c.1814C > G p.S605* c.534 + 1G > T	Het Het	AR	P (PVS1, PM3_Strong PM2_Supporting) P (PVS1, PM3_VeryStrong,PM2_Supporting)	Paternal (het) Maternal (het)	Bardet-biedl syndrome 2	TP
27	Short long bones, bowing of the long bones, abnormality of calvarial morphology, intrauterine growth retardation, coarctation of aorta	FAM111A	c.1020_1022del p.S343del	Het	AD	P (PS2_VeryStrong, PM4 PM2_Supporting)	Unknown	Gracile bone dysplasia Kenny-caffey syndrome, type 2	TP
28	Abnormality of the vertebral column, dilation of lateral ventricles	KDM1A	c.1167 + 1G > A	Het	AD	P (PVS1,PM2_Supporting)	Unknown	Cleft palate, psychomotor retardation, and distinctive facial features	TP
29	Left femur curvature	*COL1A1*	c.1615-1G > T	Het	AD	P (PVS1 PS4_Supporting,PM2_Supporting)	Paternal (het)	Osteogenesis imperfecta, type II, osteogenesis imperfecta, type III	TP
30	Right ventricular double outlet, ventricular septal defect, pulmonary stenosis, absence of pulmonary valve	*PEX10*	c.814_815del p.L272Vfs*66 c.928T > C p.C310R	Het Het	AR	P (PVS1, PS4,PM2_Supporting)	Paternal (het) Maternal (het)	Peroxisome biogenesis disorder 6A (zellweger), peroxisome biogenesis disorder 6B	Intrauterine fetal death
31	Intrauterine growth retardation, subependymal cysts, cerebellar hypoplasia, bilateral talipes equinovarus	*RARS2*	c.474_477del p.E159Lfs*2 c.1564G > A p.V522I	Het Het	AR	P (PVS1, PP1 PM2_Supporting) P (PM3_Strong,PP1,PM2_Supporting)	Maternal (het) Paternal (het)	Pontocerebellar hypoplasia, type 6	Intrauterine fetal death
32	Omphalocele, absence of left limb	*SDHB*	c.725G > A p.R242H	Het	AD	P (PS3+PS4_M + PM2_P + PM5_S + PP3)	Maternal (het)	Hereditary paraganglioma-pheochromocytoma syndrome	TP
33	Bipedal cleft deformity, left hand cleft deformity	*TP63*	c.728G > A p.R243Q	Het	AD	P (PS2+PS3_P + PS4_M + PM1 + PM2 + PM5 + PP1 + PP3)	Unknown	Hand-foot cleft type 4	Intrauterine fetal death

AD, Autosomal Dominant; AR, Autosomal Recessive; P, Pathogenicity; TP, Termination of Pregnancy; WES, Whole-Exome Sequencing.

### 3.4 *De novo* pathogenic variants

Nine *de novo* pathogenic variants were identified (Table 2). Seven *de novo* variants have been identified in fetal multisystem abnormalities, including c.1750_1754dup p. V586Rfs*114 in *TSC2*, c.593G > C p. R198P in *SNAP25*, c.6547_6560del p. Y2183Pfs*14 in *KMT2D*, c.49G > A p. E17K in *AKT1*, c.1304_1305delTG p. V435Afs*15 in *EFTUD2*, c.182A > G p. D61G in *EPHB4*, and c.2590C > T p. R864W in *PTPN11*. The *de novo* variant c.541C > Tp. R181C of *DNMT3A* was found in fetal skeleton deformities. One *de novo CREBBP* mutation, c.4614delC p. Y1539Ifs*11, was identified in fetuses with craniofacial deformity. All variants identified in these cases are *de novo*, meaning they were present in the fetus but absent in both biological parents. This is the “novel” aspect of the variants to these families. These *de novo* variants are located within known, established disease-associated genes.

### 3.5 Incidental finding

An incidental gene variant was detected and associated with conditions unrelated or partially related to the fetal phenotype. The intrauterine ultrasound phenotype in this case corresponded to omphalocele, and the left limb was absent. WES detected a heterozygous variation in *SDHB* c.725G > A (p.R242H) inherited from the mother. *SDHB* is associated with hereditary paraganglioma/pheochromocytoma (PGL/PCC) syndrome, which is inherited in an AD manner. The clinical manifestations of PGL/PCC syndrome vary, and the clinical phenotypes include paraganglioma and its complications, pheochromocytoma, and multiple organ dysfunction associated with catecholamine accumulation. The *SDHB* gene variant is considered an incidental finding because the associated clinical features are difficult to detect using prenatal ultrasound. The pathogenic SDHB variant detected here was incidental and could not explain fetal anomalies. We kept this case as incidental genomic findings are ubiquitous in prenatal testing; this example underscores vital familial tumor screening and genetic counseling considerations.

### 3.6 Pregnancy outcome and postnatal follow-up

Of the 179 pregnant women, 60 terminated their pregnancies due to severe fetal malformation or genetic abnormalities, 19 fetuses died *in utero*, eight had an abnormal phenotype after birth, and 92 had a normal phenotype (Figure 3). Among the 33 patients with P/LP variants, 19 women terminated their pregnancies, 11 fetuses died *in utero*, and the remaining women chose to continue their pregnancies after genetic counseling and careful consideration, of which one fetus had an abnormal phenotype (Table 2). Among the 146 patients with no P/LP variants, 41 experienced terminations due to severe fetal malformation, 8 fetuses died *in utero*, 7 had phenotypic abnormalities after birth (Table 3), and 90 were phenotypically normal after birth.

**FIGURE 3:**
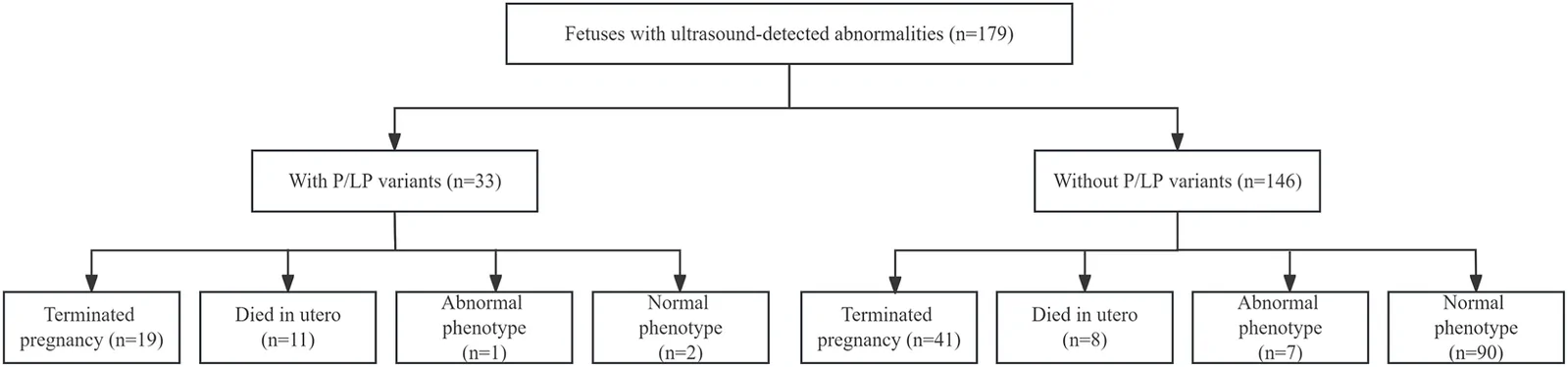
Pregnancy outcomes according to WES status (with P/LP variants versus without P/LP variants).

**TABLE 3:** Fetuses with abnormal ultrasound findings and no P/LP variants.

Case	Ultrasound phenotype	Sex	Follow-up age	Postnatal phenotype
1	Intrauterine growth retardation, oligohydria	Girl	4 months	Intellectual disability
2	Pulmonary valve atresia, patent foramen ovale, patent ductus arteriosus, pulmonary hypertension	Boy	4 years	Intellectual disability
3	Intrauterine growth retardation	Boy	2 months	Epilepsy
4	Intrauterine growth retardation	Boy	5 months	Retinitis pigmentosa, hyperglycemia
5	Hydrocephaly	Girl	2 months	Cerebral palsy
6	Ventriculomegaly	Girl	5 years	Dysgnosia, epilepsy, intellectual disability
7	Mild macrogyri	Girl	3 years	Intellectual disability

### 3.7 Diagnostic yield

For the calculation of diagnostic yield, cases were categorized based on the detection of P/LP variants. Cases in which one or more such P/LP variants were identified were counted toward the positive diagnostic yield. All other cases, regardless of other findings, were not counted as diagnostic. Applying this classification to our cohort of 179 cases yielded the following: 33 cases with P/LP variants and 146 cases without P/LP variants. The corresponding diagnostic yield was 33/179 (18.44%). More specifically, the primary diagnostic yield‑that is, the proportion of cases in which a genetic finding directly explained the primary clinical indication‑was 17.88% (32/179). When including one additional case with an incidental finding of a clinically significant SDHB variant, the overall proportion of individuals receiving a clinically actionable result increased to 18.44% (33/179). Although this SDHB variant was not related to the primary diagnostic indication, it was nevertheless considered clinically significant and was managed accordingly.

## 4 Discussion

Congenital developmental abnormalities in fetuses are often associated with genetic diseases. Disease inheritance is broadly affected by CNVs, chromosomal abnormalities, and other genetic mutations. Although CMA has the advantages of high throughput, high resolution, and automated detection, it cannot detect single-gene mutations (< 100 bp). By applying WES, a commonly used high-throughput next-generation sequencing platform (Jiang et al., 2025), this retrospective analysis identified 41 pathogenic variants in 34 genes in 33 of 179 fetuses with ultrasound abnormalities. In this study, the phenotype-explanatory diagnostic yield of WES was 17.88% (32/179). One incidental pathogenic variant irrelevant to fetal ultrasonic phenotypes was further detected, leading to an overall P/LP detection rate of 18.44% (33/179). These preliminary findings support the clinical application of prenatal WES for evaluating genotype–phenotype profiles during early pregnancy care.

Recent studies have shown that WES detects single-gene mutations in fetuses with ultrasound abnormalities, increasing the diagnosis rate by 8.5%–10% (Petrovski et al., 2019; Yates et al., 2017; Zhou et al., 2021; Fu et al., 2018; Daum et al., 2019). In this retrospective study, WES yielded a diagnostic rate of 18.44% (33/179). Differences in molecular diagnostic yields across published prenatal WES cohorts are driven by multiple methodological and clinical variables, including varying participant inclusion thresholds, variable proportions of fetuses with isolated versus multisystem structural anomalies, and inconsistent grouping rules for variants of uncertain significance when calculating positive detection rates. Our cohort primarily enrolls pregnancies with fetal structural abnormalities and excludes VUS-only cases from definitive positive outcomes, which collectively contributes to the distinct diagnostic rate observed in our analysis. The observed increase likely reflects key methodological differences, including our study’s specific cohort inclusion criteria, potential enrichment for multisystem or complex fetal phenotypes, and the reporting of significant incidental findings (Jelin and Vora, 2018). Additionally, our reported diagnostic yield is based exclusively on pathogenic and likely pathogenic variants, excluding variants of uncertain significance from the calculation. In this study, of the WES-detected fetuses (27 of the 33 fetuses whose parents consented to family verification, with unknown genetic modes), 18 (66.67%) genotypes were inherited from the parents, and the risk of recurrence was high during the second pregnancy. This suggests that genetics may be the dominant factor in fetuses with ultrasound abnormalities, highlighting the importance of early genetic counseling and carrier screening in pregnant women. We observed nine cases of *de novo* pathogenic variants, seven of which were detected in fetal multisystem abnormalities, one in skeletal abnormalities, and one in craniofacial deformities, suggesting that *de novo* pathogenic variants may also play important roles in structural abnormalities. Therefore, WES can provide a genetic diagnosis for fetal ultrasound abnormalities and inform decision making in genetic counseling, reproductive guidance, and personalized treatment.

Fetal craniofacial deformities were the most common among the single-system abnormalities detected using WES, with only a few cases included, which could introduce bias in the results. This result is based on a very small sample size and is associated with a wide 95% confidence interval of 9.40%–99.20%. A fetus with cleft lip, cleft palate, and orofacial cleft was associated with a pathogenic mutation in *CREBBP,* which is also associated with Rubinstein-Taybi syndrome (Eser et al., 2017), a multiple congenital malformation syndrome characterized by distinctive facial features, wide and angular thumbs/toes, short stature, and moderate-to-severe intellectual disability. The WES detection rate for skeletal system and congenital heart defects was second only to that of craniofacial deformities, consistent with previous research results (Zhou et al., 2021; Huang Y. et al., 2023). One fetus presented with tetralogy of Fallot, tricuspid regurgitation, mitral regurgitation, and ventricular hypertrophy, and WES indicated that pericardial effusion was associated with compound heterozygous variants of PLD1 genes, which are also associated with developmental heart valve defects (Zhu et al., 2024). WES in fetuses with skeletal dysplasia has shown a strong relationship with *FGFR3* mutations (Petrovski et al., 2019; Lord et al., 2019). In this study, the intrauterine ultrasound phenotypes of one fetus were short, long bones, limb undergrowth, and intrauterine growth retardation. WES revealed that *FGFR3* heterozygous mutations were related to thanatophoric dysplasia and achondroplasia. In a fetus presenting with inferior vermis hypoplasia and hydrocephalus, WES detected a heterozygous variation in the *COL4A1* gene inherited from the father, which is related to brain small vessel disease (BSVD) with or without ocular anomalies (England et al., 2021). BSVD is an AD genetic disorder characterized by variable penetrance and expression patterns, as evidenced by the absence of significant clinical manifestations in the father. The ultrasound phenotype of one fetus exhibited a polycystic kidney, which WES was associated with a heterozygous mutation in the pathogenic *PKD1* gene. The ultrasound phenotype and genetic results were like those reported by Shuster et al. (Shuster et al., 2019). The present and previous findings highlight the applicability of WES for complementing prenatal imaging. In this study, fetuses with omphalocele and missing left limbs were found to carry maternal heterozygous variants of the *SDHB* gene, which are also associated with accessory neuroma and pheochromocytoma in young adulthood (Jochmanova et al., 2017). The combination of ultrasound phenotyping and WES can minimize false detection of fetal abnormalities and supplement CMA in clarifying prenatal diagnosis.

The pregnancy outcomes of 179 fetuses were tracked and followed up. Among them, one case with a heterozygous variation in the *COL4A1* gene was characterized by hydrocephalus and inferior vermis hypoplasia on intrauterine ultrasound, and intellectual disability was found on postnatal follow-up. Phenotypic abnormalities occurred after birth in seven fetuses not detected by WES, three of which had central nervous system abnormalities, three had skeletal abnormalities, and one had a congenital heart defect. This suggests that fetuses with central nervous system abnormalities on intrauterine ultrasound are more likely to exhibit clinical phenotypes after birth. The fetuses with thickened nuchal translucency, polyhydramnios, digestive system malformation, and abnormal urinary system had normal phenotypes during the initial follow-up. This shows that WES has a greater guiding value in the genetic diagnosis of fetal ultrasound abnormalities and in determining whether to terminate pregnancy. The identification of a somatic mosaic AKT1 variant in a fetus with features suggestive of *Proteus* syndrome is a particularly instructive finding. It demonstrates that high-level somatic mosaicism detectable in amniocytes can underlie severe, asymmetric overgrowth syndromes. This case reinforces that prenatal WES, especially when a tissue sample like amniotic fluid (a mix of fetal cells) is analyzed, must be interpreted with the possibility of mosaicism in mind. It validates the utility of WES in such complex cases where the phenotype may not point to a single-gene disorder and underscores the importance of variant allele fraction analysis in the bioinformatic pipeline. For fetuses in whom no P/LP variants were identified yet who subsequently manifested abnormal postnatal phenotypes, further genetic evaluation remains paramount. A result without reported P/LP variants does not definitively exclude all genetic etiologies. This situation underscores a critical ‘grey area’ between a non-diagnostic test result and a true genetic resolution. Important considerations include variants not captured by the testing methodology (e.g., non-coding, structural, or epigenetic alterations), the possibility of disorders with oligogenic, non-genetic, or multifactorial causes, and the role of VUS.

The identification of the pathogenic SDHB variant in our cohort, unrelated to the fetal indication but conferring a high risk of adult-onset pheochromocytoma, highlights a critical ethical dimension of prenatal WES. Our protocol adheres to the ethical framework for secondary findings, and written informed consent for prenatal WES explicitly included the option to receive such results. This case underscores the need for comprehensive pre-test genetic counseling to prepare parents for the possibility of actionable incidental findings. The management of such information, balancing parental autonomy, future child autonomy, and the principle of beneficence, remains a complex but essential component of responsible prenatal genomics.

This study had limitations. The results may be biased because of the small sample size. More objective clinical data should be collected to further clarify the applicability of WES for the genetic diagnosis of fetal abnormalities. Although WES can improve the detection of single genes, some detection errors may persist (Kim et al., 2023). WES detection rates have varied greatly between studies (Best et al., 2018), potentially owing to incomplete expression of fetal symptoms and limited clinical information. Further development is needed to standardize WES procedures in terms of clinical indications, relevant technical processes, data interpretation, and testing-related consultations. While most prenatal WES tests in this cohort were performed during the second trimester to guide reproductive decision-making, a subset (3.35%) was conducted in the third trimester (≥ 28 weeks of gestation). In these later cases, the primary clinical objectives shifted toward guiding perinatal management, optimizing delivery planning, and facilitating immediate postnatal care for the newborn. Although the prenatal application of WES remains challenging, further research in this field is important to improve the interpretation and clinical indications of WES data and inform genetic counseling and care of pregnant families. We acknowledge the absence of segregation data in a subset of cases where biological parents declined further analysis. This highlights a practical constraint in clinical research that future efforts aim to address. The mean postnatal follow-up period in our cohort was 6 months. This duration is currently insufficient for a comprehensive assessment of neurodevelopmental outcomes, as many such disorders manifest beyond infancy or early childhood. Our preliminary outcome assessments are therefore focused on growth, congenital anomaly confirmation, and early neurological signs. Finally, statistical analysis and outcome reporting in our study were restricted exclusively to P/LP variants. While all identified VUS were recorded in individual clinical laboratory reports for prenatal genetic consultation, we did not systematically extract, collate or summarize VUS data for the current retrospective dataset. Accordingly, the calculated diagnostic yield in our research only accounts for P/LP variants. Subsequent cohort studies integrating thorough VUS profiling will facilitate consistent comparisons with previously published prenatal cohorts.

This cohort study provides practical translational evidence for exome genetic screening. First, our stratified analysis standardized reference information for genetic counselors to deliver precise pregnancy risk assessments, especially for families carrying asymptomatic parental pathogenic gene with reduced penetrance. Second, we clarified the limitations of WES, epigenetic abnormalities and low-level mosaicism as causes of adverse fetal outcomes, which reminds clinicians to adopt multi-platform combined genetic testing for high-risk fetuses with structural ultrasound anomalies. Third, the observed incidental secondary genetic findings such as the SDHB variant emphasize the necessity of standardized postnatal surveillance and extended familial genetic screening after prenatal sequencing. Collectively, these findings optimize the clinical workflow of prenatal genomic diagnosis and help reduce overinterpretation or underappreciation of fetal genetic variants during prenatal counseling.

## 5 Conclusion

The conclusion has been updated to reflect the P/LP variants yield of 17.88% (32/179) as the primary diagnostic finding. The ultrasound phenotypes of most fetuses were consistent with the clinical phenotypes associated with disease-causing genes. By combining molecular testing with prenatal ultrasound phenotypes, clinicians can identify the underlying genetic cause, assess the risk of recurrence, and provide more comprehensive information to affected parents. This should ultimately promote the development of personalized management plans for families at a higher risk of recurrence.

## Data Availability

The datasets presented in this article are not readily available because of ethical and privacy restrictions. All core aggregated data supporting the conclusions of this study have been fully included within the manuscript, supplementary tables and figures. All variant frequencies, patient phenotypic information and key analytical results required to evaluate our findings are completely available in the article. Requests to the de‐identified raw data can be available upon reasonable request to the corresponding author, subject to institutional ethics approval and data‐use agreement.
